# Peritumoural neutrophils negatively regulate adaptive immunity via the PD-L1/PD-1 signalling pathway in hepatocellular carcinoma

**DOI:** 10.1186/s13046-015-0256-0

**Published:** 2015-11-18

**Authors:** Gaixia He, Henghui Zhang, Jinxue Zhou, Beibei Wang, Yanhui Chen, Yaxian Kong, Xingwang Xie, Xueyan Wang, Ran Fei, Lai Wei, Hongsong Chen, Hui Zeng

**Affiliations:** Peking University People’s Hospital, Peking University Hepatology Institute, No.11 Xizhimen South Street, Beijing, 100044 China; Beijing Key Laboratory of Hepatitis C and Immunotherapy for Liver Diseases, Beijing, 100044 China; Department of Hepatobiliary and Pancreatic Surgery, Henan Tumour Hospital, Zhengzhou, Henan 450008 China; Institute of Infectious Diseases, Beijing Ditan Hospital, Capital Medical University, Beijing, 100015 China; Beijing Key Laboratory of Emerging Infectious Diseases, Beijing, 100015 China

**Keywords:** Neutrophils, Hepatocellular carcinoma, Predictive Value, Programmed death ligand 1

## Abstract

**Background:**

PD-L1 expression on neutrophils contributes to the impaired immune response in infectious disease, but the detailed role of PD-L1 expression on neutrophils in HCC remains unclear.

**Methods:**

We investigated the phenotype and morphology of neutrophils infiltrated in tumour tissues from both patients with HCC and hepatoma-bearing mice.

**Results:**

We found that neutrophils dominantly infiltrated in the peritumoural region. The neutrophil-to-T cell ratio (NLR) was higher in peritumoural tissue than that in the intratumoural tissue and was negatively correlated with the overall survival of patients with HCC. Infiltrating neutrophils displayed a phenotype of higher frequency of programmed cell death ligand 1 (PD-L1) positive neutrophils. The ratio of PD-L1^+^ neutrophils-to-PD-1^+^ T cells was higher in peritumoural tissue and better predicted the disease-free survival of patients with HCC. We further confirmed a higher frequency of PD-L1^+^ neutrophils and PD-1^+^ T cells in hepatoma-bearing mice. Functionally, the PD-L1^+^ neutrophils from patients with HCC effectively suppressed the proliferation and activation of T cells, which could be partially reversed by the blockade of PD-L1.

**Conclusions:**

Our results indicate that the tumour microenvironment induces impaired antitumour immunity via the modulation of PD-L1 expression on tumour infiltrating neutrophils.

**Electronic supplementary material:**

The online version of this article (doi:10.1186/s13046-015-0256-0) contains supplementary material, which is available to authorized users.

## Background

Hepatocellular carcinoma (HCC) remains one of the most malignant cancers, as the incidence and mortality of this cancer continues to increase each year [1]. The prognosis of patients with HCC remains poor due to the high rate of recurrence and metastasis. HCC often develops from long-term chronic inflammation that is primarily caused by HBV/HCV infection or alcoholic hepatitis, which suggests the role of crosstalk between inflammation and tumours in the development of HCC [2].

Tumour-associated inflammation has been considered one of the hallmarks of cancer progression [3]. Evidence indicates an impaired anti-tumour response within the hepatoma microenvironment due to various immune suppressive elements, including regulatory T cells (Tregs) [4], tumour-associated macrophages (TAMs) [5], tumour-associated fibroblasts (TAFs) [6] and tumour-associated neutrophils (TANs) [7, 8]. Recently, TANs have been considered a new target for cancer immunotherapy. As one of the most abundant types of immune cells, neutrophils in the tumour microenvironment participate in nearly every step of tumour progression, which involves the following: the suppression of adaptive immunity, the promotion of neoangiogenesis and lymphangiogenesis, the remodelling of the extracellular matrix, the promotion of invasion and metastasis, and the inhibition of specific antigen-induced anti-tumour T cell responses, as have been previously reviewed [9]. High levels of tumour-infiltrating neutrophils have been shown to be associated with advanced disease and poor clinical outcome in patients with cancer [10, 11]. In addition, an elevated preoperative neutrophil-to-lymphocyte ratio (NLR) in both blood and tissue has been shown to negatively correlate with the prognosis of patients with HCC [12, 13], which indicates that a disturbed balance between neutrophils and T lymphocytes in individuals with cancer could affect both local and systemic immune responses. An understanding of the underlying mechanisms of the correlation between a high NLR and poor survival of patients with tumours is significant for targeted cancer therapy.

Among a number of mechanisms that mediate T cell anergy and tumour immune evasion in patients with HCC, Program death ligand 1 (PD-L1) and Program death (PD-1) have emerged as central players [14, 15]. It is exciting that antibodies against PD-L1 and PD-1 have been under preclinical and clinical development for cancer therapy [16–24]. Overexpression of PD-L1, however, contributes to evasion of the immune system. For example, in the case of HCC, PD-L1 was shown to be overexpressed on tumour cells [14], Kupffer cells [25] and TAMs [5], which induced apoptosis of T cells and immune tolerance. The blockade of the PD-L1/PD-1 interaction could reduce cancer development and enhance the T cell-mediated antitumour response. All of this evidence indicates the critical role of the PD-L1/PD-1 signalling pathway in the development of HCC.

Neutrophils have been considered non-professional antigen presenting cells (APCs). Recent studies have demonstrated the expression of co-inhibitory molecules, such as PD-L1, following in vitro exposure to cytokines [26] or after stimulation by LPS or toll-like receptors (TLRs) [27]. PD-L1 expression on neutrophils was also increased in vivo in patients with active tuberculosis (TB) or HIV infection while it was decreased in patients who received anti-TB therapy [28] or anti-viral treatment [27]. These reports suggested the functional role of PD-L1 expression on neutrophils. Previous studies predominantly focused on tumour-infiltrating neutrophils, but the role of neutrophils that infiltrate the peritumoural edge remains unclear. Here, we investigated the phenotype and function of neutrophils from tumour tissues and from tissues along the edge of the tumour in patients with HCC who underwent surgical resection.

In this study, we revealed an accumulation of PD-L1^+^ neutrophils in the peritumoural region of patients with HCC. A lower level of neutrophil infiltration in peritumoural tissue predicted a better overall survival in patients with HCC. A decreased PD-L1^+^neutrophil/PD-1^+^ T cell ratio in peritumoural tissues was closely correlated with prolonged recurrence-free survival (RFS) of patients with HCC. Furthermore, tumour-derived factors, including GM-CSF and TNF-α, contributed to PD-L1 expression on neutrophils in patients with HCC. The levels of tumour-derived soluble factors, including GM-CSF, TNF-α, G-CSF and MCP-1, are higher than those in the tumour site. Taken together, we propose an alternative explanation that higher level of neutrophils in peritumoural tissues was negatively correlated with the prognosis in hepatocellular carcinoma.

## Methods

### Study subjects and cell lines

All 149 patients with HCC from Guilin Medical College Affiliated Hospital were enrolled according the following criteria: a) the pathology of each patient was confirmed; b) patients did not exhibit signs of distant metastasis and had not received anticancer therapy prior to surgery. The tumour stage was determined according to the 6th edition tumour-node-metastasis (TNM) staging system [29]. Data were censored at the last follow-up for patients without recurrence or death. The recurrence-free survival time (RFS) was defined as the interval between the time of surgery to the time of recurrence, while the overall survival time (OS) was defined as the interval between the time of surgery and death. Healthy donors (*n* = 32) were patients at Beijing hospital who had received a physical examination. All samples were collected in accordance with local ethical guidelines. The ethics committee of Peking University People’s Hospital approved the protocol of all written informed consent. Conventional clinicopathologic variables and treatment modalities that were given after resection are detailed in Additional file 1: Table S1.

With regards to the animal experiment, 6–8-week-old female BALB/c mice were used. All animal protocols were approved by the Review Board of Peking University People’s Hospital. All animals were provided by China Academy of Military Medical Science and bred in specific pathogen-free (SPF) animal facilities in our hospital. H22 cells (hepatoma cell line) were injected in BALB/c background mice subcutaneously at 4 × 10^5^ per mouse in 100 μl sterile saline to obtain tumour-bearing mice. Mice in the control group received an equivalent volume of sterile saline.

For in vitro experiments, human HCC cell line of HepG2 and MHCC-97H were used. LX-2 cell, which was established for hepatic stellate cells, was used to mimic the liver microenvironment.

### Sample collection and isolation of cells

Peripheral blood- and tissue-infiltrated cells were collected and isolated as previously described. Tissue-infiltrated lymphocytes were obtained by density gradient centrifugation with 40 % Percoll and 80 % Percoll (GE Healthcare Bio-Sciences AB, Sweden). For functional analysis, human neutrophils were isolated and then purified according to CD16 and CD66b coexpression by the BD Aria II Cell Sorting System (BD Bioscience, USA). CD4^+^ T cells and CD8^+^ T cells were enriched by a magnetic cell separation system (Miltenyi Biotec, Germany). The purity was above 95 % and the viability was above 93 %, as evaluated by flow cytometry and 0.4 % trypan blue (Gibco BRL) staining, respectively.

### Tissue microarray (TMA) construction and Immunohistochemistry (IHC)

Biopsy samples were fixed in 10 % formalin and paraffin-embedded. Then, the samples were cut into 5-μm-thick sections. Immunohistochemistry was performed according to the appropriate protocols as previously described [14]. The following antibodies were used as primary antibodies: anti-human CD66b (Anaspec, CA, USA), anti-human CD3 (Gene Tech, Shanghai, China), anti-human PD-L1 and anti-human PD-1 (BD Bioscience, San Jose, USA). A mouse anti-human CD66b monoclonal antibody was used to identify neutrophils (1:50), and rabbit anti-human CD3 monoclonal antibody was used to identify T cells (1:100). A Real Envision Detection Kit (Gene Tech, Shanghai, China) was used for detection. Quantification of immune-cell infiltration was then determined.

### Flow cytometric analysis

For flow cytometry, the following antibodies for the analysis of human samples were used: FITC-conjugated anti-CD16, PE-conjugated anti-PD-L1, PerCP-conjugated anti- CD45, APC-conjugated anti-CD11b, APC-conjugated anti-CD14, FITC-conjugated anti-CD3, PE-conjugated anti-CD8, PerCP-conjugated anti-CD4 and APC-conjugated anti-PD-1. All human antibodies were purchased from BD Bioscience (San Jose, USA). Antibodies used in the mouse experiments were purchased from eBioscience (San Diego, CA) as follows: FITC-conjugated anti-Gr-1, PE-conjugated anti-PD-L1, PerCP-conjugated anti-CD11b, APC-conjugated anti-CD48, APC-conjugated anti-CD8, FITC-conjugated anti-CD3, PE-conjugated anti-PD-1, and PerCP-conjugated anti-CD4. Data were analysed with FlowJo5.6.7 (Tree star, Inc., Ashland, OR, USA).

### Cytokines analysis by Luminex technology

Cytokines in the culture supernatant and tissue lysates were evaluated with the Luminex200 System (Merck Millipore, Darmstadt, Germany). The detection kit includes the following cytokines: G-CSF, GM-CSF, IL-6, IL-1β, IL-8, IL10, IL-17, TNF-α, IFN-γ, TGF-β, MCP-1, SDF-1 and MIP-1α. Briefly, hepatoma cell lines were maintained at 70 % cell confluence in Dulbecco’s Modified Eagle’s Medium (DMEM) supplemented with 10 % FBS. After 48 h, the supernatant was collected and centrifuged to deplete contaminated cells. In some experiments, the tumour cells were pretreated with LX-2 supernatant for 24 h. Tumour tissues were preserved at −80 °C until use. We used 500 μl of tissue lysis buffer for 30 mg of tumour tissue, peritumoural tissue and adjacent non-tumour tissue. Lysis buffer was applied for the analysis of cytokines.

### In vitro culture assay

Tumour culture supernatant (TSN) was obtained as described above. Briefly, hepatoma cell lines were maintained at 70 % cell confluence in DMEM supplemented with 10 % FBS. After 48 h, the supernatant was collected and centrifuged to remove the contaminated cells. Purified neutrophils from healthy donors were then exposed to culture medium with TSN (50 %, v/v) from hepatoma cell lines or cytokines including TNF-α (200 U/ml) and GM-CSF (20 ng/ml). Neutrophils cultured in medium alone served as the control group.

### RNA extraction and quantitative real-time PCR (qRT-PCR)

Total RNA of the neutrophils was extracted with Trizol reagent (Invitrogen, USA). cDNA was synthesized with High Capacity RNA-to-cDNA Kit (Applied Biosystems, USA). qPCR was performed with a LightCycler 480 (Roche Molecular Biochemicals, Mannheim, Germany) and SYBR Select Master Mix (Applied Biosystems, USA). The specific primer sequences for human PD-L1 and GAPDH (for normalisation) are listed as follows: PD-L1, forward 5’-TGGCATTTGCTGAACGCATTT-3’, reverse 5’-TGCAGCCAGGTCTAATTGTTTT-3’ and GAPDH, forward 5’- ACAACTTTGGTATCGTGGAAGG-3’, reverse 5’- GCCATCACGCCACAGTTTC -3’, respectively. All of the above studies were performed according to the manufacturer’s protocol.

### Statistical analysis

All statistical analysis was performed with SPSS 16.0 (SPSS Inc., Chicago, IL, USA). Differences in the immunostaining of the cells between the subgroups were determined by the paired samples *t*-Test. Correlations between parameters were assessed with the Pearson correlation analysis and linear regression analysis. Univariate and multivariate analysis were performed with the Kaplan–Meier method as well as the Cox proportional hazards regression model and were compared with the log-rank test. Values of *P <* 0.05 were considered statistically significant between the different groups. The in vitro studies were performed independently in at least three separate experiments.

## Results

### A high neutrophil-to-lymphocyte ratio in peritumoural tissues correlated with poor prognosis in patients with HCC

**Fig. 1:**
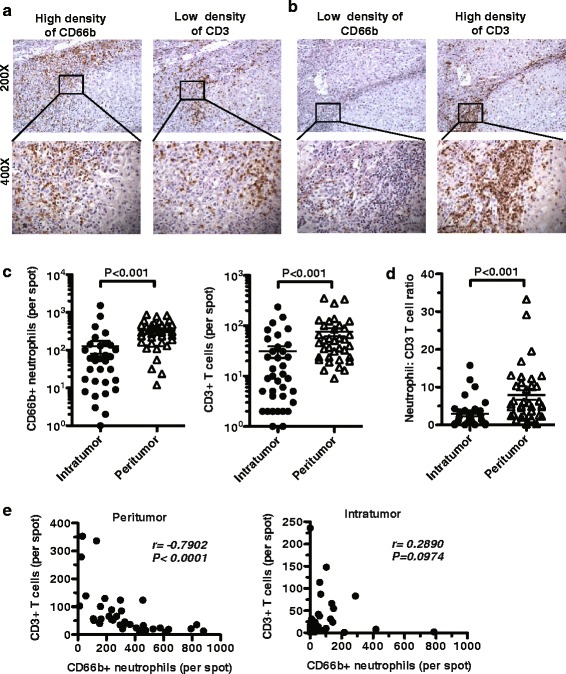
Neutrophils predominantly infiltrated in the peritumoural tissue in patients with HCC. **a**, **b** Representative images were shown for CD66b and CD3 immunohistochemical staining of tumour tissues from patients with HCC. A high density of CD66b^+^ neutrophils is correlated with a low density of CD3^+^ T cells in the tumour tissue. **c** Infiltrating CD66b^+^ neutrophils and CD3^+^ T cells in intratumoural tissue and peritumoural tissues that were analysed by paired *t*-Test (*P <* 0.001 for both). **d** The neutrophil/T cell ratio (NLR) in intratumoural tissue and peritumoural tissues that were analysed by paired *t*-Test (*P <* 0.001 for both). **e** Correlation of the number of CD66b^+^ neutrophils and CD3^+^ T cells in peritumoural tissues and intratumoural tissues in patients with HCC as analyzed by Pearson correlation analysis and linear regression analysis (*r* = −0.7902, *P <* 0.001 and *r* = 0.2890, *P* = 0.0974, respectively)

To investigate the distribution of neutrophils and T lymphocytes within the tissue, we performed immunohistochemical staining for CD66 and CD3 in tumour samples from 149 patients with HCC. Strikingly, compared with the tumour nests, the peritumoural tissues contained a higher number of CD66b^+^ neutrophils as well as CD3^+^ T cells (*P <* 0.001, Fig. 1a, b and c). Moreover, the regions with a higher density of CD66b^+^ neutrophils usually showed a lower number of CD3^+^ T cells, and similarly, the regions with a lower density of CD66b^+^ neutrophils usually accumulated abundant CD3^+^ T cells (Fig. 1a, b). This suggested that the number of neutrophils might be negatively correlated with CD3^+^ T cells in the peritumoural region. We calculated the CD66b^+^ neutrophil-to-CD3^+^ T cell ratio (NLR) in the peritumoural tissue (pNLR) and in the intratumoural tissue (iNLR), and found that the pNLR was significantly higher than the iNLR (pNLR = 5.600 ± 0.589 and iNLR = 2.118 ± 1.035, Fig. 1d,*n* = 37). Furthermore, a Pearson correlation coefficient analysis confirmed the hypothesis that the number of infiltrated neutrophils was negatively correlated with the number of lymphocytes in the peritumoural regions (Fig. 1e, r = −0.7902, *P* < 0.001, *n* = 37) but not in the intratumoural regions (Fig. 1e, r = 0.2890, *P* = 0.0974, *n* = 37).

**Fig. 2:**
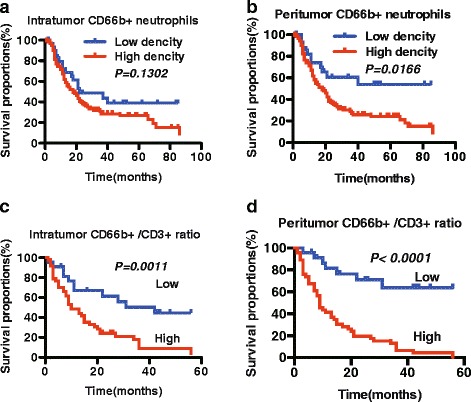
A high level of neutrophil infiltration correlates with poor prognosis of patients with HCC. Neutrophil infiltration in peritumoural tissue (**b**) but not in tumoural tissue (**a**) predicts overall survival. The NLR in both peritumoural tissues and tumour tissues in patients with HCC is divided into two groups (low, NLR < 5.35; high, NLR>/=5.35), according to the median value of the pNLR at each specific site. The NLR in both peritumoural tissue (**d**) and tumour tissue (**c**) could predict a poorer overall survival in patients with HCC. The Kaplan–Meier method estimates of the probability graphs of the survival of the patients and the survival time were calculated from the time of surgery until the death of the patients. The log-rank test was used for comparisons between the two groups

Based on the above observations, we tested the correlation between the infiltration of neutrophils in different regions and the clinical prognosis of patients with HCC. We followed 149 patients with HCC for 90 months. According to the median value of the number of CD66b^+^ neutrophils in peritumoural tissue, >305 cells/field under the microscope was considered high (*n* =120). The univariate analysis revealed that an increase in the number of intratumoural neutrophils was not significantly associated with patients’ postoperative survival (Fig. 2a, *P* = 0.1302). In contrast, the patients with increased peritumoural CD66b^+^ neutrophils showed a decreased overall survival rate (Fig. 2b. *P* = 0.0166). Next, after we performed immunohistochemistry for the double-labelling of CD66b and CD3, we divided the 69 patients with HCC into two groups according to the median value of the NLR. The postoperative survival time of individuals in the lower CD66b^+^/CD3^+^ ratio group was significantly longer than that of individuals in the high NLR group (low, NLR < 5.35, *n* = 46, median survival 42.0 months; high, NLR ≥ 5.35, *n* = 23, median survival 10.0 months). We found that both high pNLR (Fig. 2b, *P* < 0.0001) and high iNLR (Fig. 2d, *P* = 0.0011) were significantly correlated with poor overall survival (OS). In addition, the pNLR may be better than iNLR for the prediction of OS in patients with HCC.

### Overexpression of PD-L1 on neutrophils predicts poorer survival in patients with HCC

**Fig. 3:**
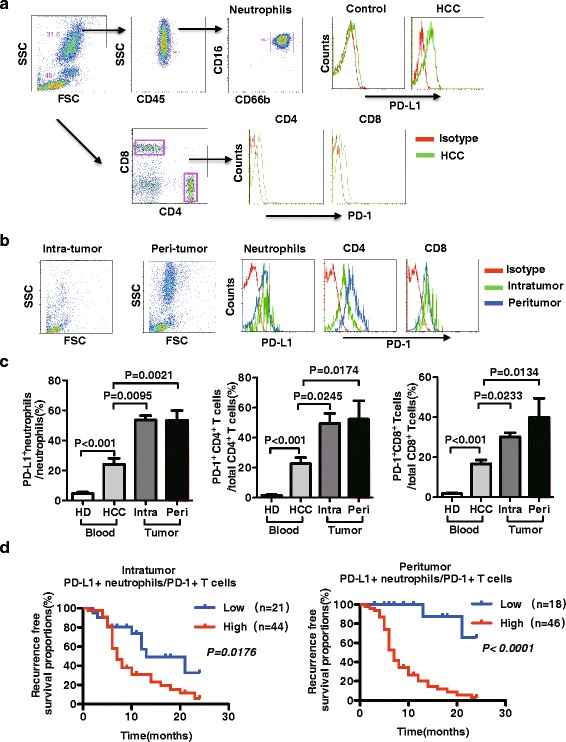
PD-L1 expression was up-regulated on neutrophils in patients with HCC, and was negatively correlated with patients’ survival. **a** Representative PD-L1 expression on neutrophils and PD-1 expression on CD4^+^/CD8^+^ T cells from the blood of HD and HCC patients. **b** Tissue-infiltrated neutrophils from tumour tissue and peritumoural tissue were isolated and analyzed by FACS analysis. **c** Calculative percentage of PD-L1^+^ neutrophils, PD-1^+^ CD4 T cells and PD-1^+^ CD8 T cells in circulation of HD and HCC and in intra-tumoural and peri-tumoural tissue, by gating neutrophils, CD4^+^ T cells and CD8^+^ T cells. **d** Kaplan–Meier estimates of the RFS in patients with high (red line) and low (blue line) PD-L1^+^ neutrophil/PD-1^+^ T cell ratios in peritumoural (*P* < 0.0001, *n* = 64) and intratumoural tissues (*P* = 0.176, *n* = 65). HD, healthy donors

Next, we assessed the expression of PD-L1 on circulating neutrophils and on tumour-infiltrating neutrophils from patients with HCC. Compared with healthy donors (HDs), the surface expression of PD-L1 on circulating neutrophils was higher in patients with HCC (Fig. 3a). We observed increased PD-1 expression on CD4^+^ T and CD8^+^ T cells (Fig. 3a). Moreover, the frequency of PD-L1 expressing neutrophils increased in both intratumoural and peritumoural tissues compared with circulating neutrophils (Fig. 3a,*n* = 32 for HD, *n* = 27 for HCC). And the frequency of PD-1-positve cells was also significantly increased on CD4^+^ and CD8^+^ T cells that were isolated from intratumoural and peritumoural tissues (Fig. 3a). Further, we analyzed the morphology of neutrophils that are infiltrated in tumour tissue. We indicated the individual morphological status of PD-L1^+^ and PD-L1^−^ neutrophils in tumour tissue that PD-L1^+^ neutrophils indicated segmented nuclei while the fraction of PD-L1^−^ neutrophils showed circle nuclei (Additional file 2: Figure S1).

The above findings were further confirmed by the immunohistochemical staining of tumour tissues and adjacent peritumoural tissues from a separate cohort of patients with HCC (*n* = 65 for peritumoural, *n* = 64 for intratumoural). PD-L1/PD-1 pathway is usually associated with tumour progression and early recurrence. We calculated the number of PD-L1^+^ neutrophils and the number of PD-1^+^ T cells. The PD-L1^+^ neutrophil/PD-1^+^ T cell ratio in both peritumoural and intratumoural tissues could predict the postoperative recurrence-free survival (ROS) (Fig. 3b for intratumoural, *P* = 0.0176; Fig. 3c for peritumoural, *P* < 0.0001).

### PD-L1 expression was up-regulated in hepatoma-bearing mice

**Fig. 4:**
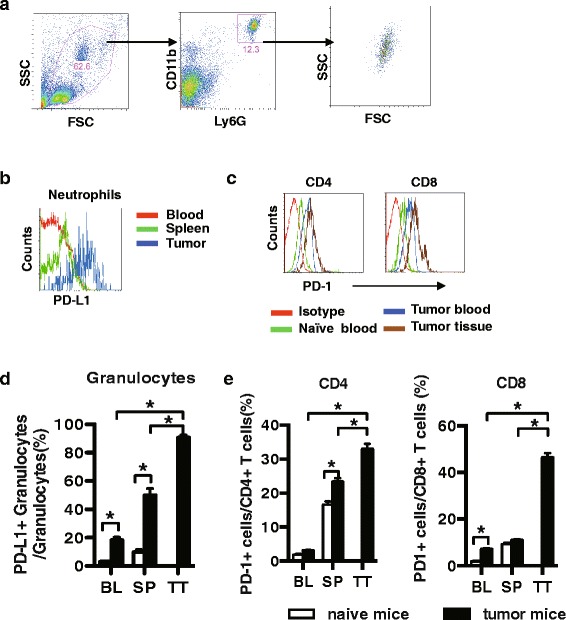
PD-L1 expression was up-regulated in hepatoma-bearing mice. **a** Neutrophils in mice were gated and analyzed according CD11b^+^Ly6G^+^. **b** Data shown was from tumour-bearing mice of PD-L1 expression on neutrophils in circulation and tissue. **c** Data shown was from tumour-bearing mice of PD-1 expression on CD4^+^ T cells and CD8^+^ T cells in circulation and tissue. **d** The percentage of PD-L1^+^ neutrophils was shown in tumour-bearing mice (n = 8). **e** PD-1 expression was increased slightly on CD4^+^ and CD8^+^ T cells in the blood and spleen of hepatoma-bearing mice vs. naïve mice (n = 8). Experiments were performed in at least three independent studies. BL, blood; SP, spleen; TT, tumour tissue. Data are presented as the mean ± SEM. *P < 0.05

To further confirm the above results from patients with HCC, we assessed the expression levels of PD-L1 on neutrophils in hepatoma-bearing mice. According to previous studies, CD11b^+^Ly-6G^+^ neutrophils were gated by flow cytometry (Fig. 4a). Two weeks after the inoculation of hepatoma cells into the mice, a significant increase in the frequency of neutrophils was observed in the bone marrow, the spleen and the peripheral blood in tumour-bearing mice (data not shown). Of note, compared with neutrophils in the blood and spleen, tumour-infiltrating neutrophils exhibited higher frequency of PD-L1^+^ cells (Fig. 4b, d). In addition, we observed increased PD-1 expression on CD4^+^ T and CD8^+^ T cells that were isolated from the spleen and tumour tissues of tumour-bearing mice compared with those isolated from naïve mice (Fig. 4c, e).

### PD-L1 expression on neutrophils was induced by the tumour microenvironment

**Fig. 5:**
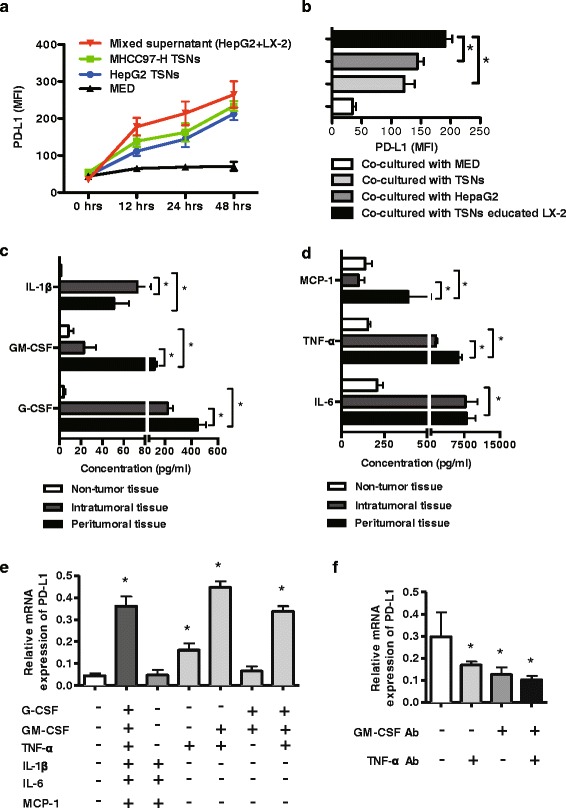
PD-L1 expression on neutrophils is induced by the cytokines of GM-CSF and TNF-α. **a** Purified neutrophils from healthy controls were exposed to conditioned medium in vitro as indicated. PD-L1 expression on neutrophils was determined at 0, 6, 24 and 48 h. **b** The mean fluorescence intensity (MFI) of PD-L1 on neutrophils is shown. **c, d** Selected cytokines and chemokines were detected by liquid chip technology in conditioned medium from normal liver cells or hepatoma cells and tissue lysis supernatants from intratumoural and peritumoural tissues. **e** Neutrophils were exposed to the indicated cytokines for 24 h. The relative expression of PD-L1 on neutrophils was detected by qRT-PCR. **f** PD-L1 expression on neutrophils was significantly reduced with specific neutralizing antibodies against GM-CSF and TNF-α compared with the control (Student’s *t*-Test)

Because inflammation and the tumour microenvironment were largely responsible for the induction of PD-L1 expression on immune cells, we wondered whether the tumour microenvironment might also induce PD-L1 expression on infiltrating neutrophils in patients with HCC. We used HCC cell lines and LX-2 cells to mimic the hepatic microenvironment in an in vitro study. We purified neutrophils from fresh blood of healthy volunteers and then cultured these neutrophils with tumour conditioned supernatant (TSNs) from the hepatoma cell lines of HepG2 and MHCC-97H or from LX-2-pretreated hepatoma cells (HepG2/LX-2 TSNs) for the indicated time points. The results showed that PD-L1 expression on neutrophils was increased in a time-dependent manner in response to TSNs or HepG2/LX-2 TSN treatment. In addition, neutrophils that were exposed to HepG2/LX-2 TSN exhibited a greater expression of PD-L1 than those neutrophils that were exposed to TSNs or control culture medium (Fig. 5a, b).

Based on the above results, the cytokines in the tumour supernatant and in the tissue lysates from tumours, peritumoural and adjacent non-tumour tissues were analysed with a Luminex 200 system as described in the materials and methods section. We observed higher levels of several cytokines, including G-CSF, IL-1β, GM-CSF, IL-6, IFN-γ, TNF-α, TGF-β, RANTES, MCP-1, SDF-1 and MIP-1α, in TSNs compared with control culture medium (Additional file 3: Figure S2). This in vitro observation was confirmed with tissue lysates from patients with HCC. The levels of cytokines and chemokines, including IL-1β, IL-6, GM-CSF, G-CSF, MCP-1 and TNF-α, were much higher in tumour tissue lysates than in non-tumour tissue. Furthermore, the levels of the cytokines GM-CSF and TNF-α, and the chemokine MCP-1 were much higher in tissue lysates from peritumoural tissues compared with tumour tissues (Fig. 5c, d). This indicates the differences in the tumour microenvironment of peritumoural tissues and that of the actual tumour itself.

The factors that lead to PD-L1 expression on neutrophils in the tumour microenvironment are not completely understood. Furthermore, PD-L1 expression on tumour cells and macrophages was up-regulated in the tumour microenvironment of HCC. Next, we tested whether the above factors contributed to the up-regulation of PD-L1 expression on neutrophils. We cultured neutrophils in the presence of the selected cytokines in vitro (Fig. 5e). Based on all of the conditions that were tested, combination of GM-CSF and TNF-α were able to dramatically enhance PD-L1 expression on neutrophils (Fig. 5e). In addition, PD-L1 expression on neutrophils was increased in response to TNF-α treatment. Furthermore, we added specific neutralizing antibodies against GM-CSF and TNF-α into the co-culture systems with TSN. Both neutralizing antibodies partially attenuated the expression of PD-L1 induced by the tumour cell culture supernatants (Fig. 5f), which indicated that GM-CSF and TNF-α in the tumour microenvironment are involved in the expression of PD-L1 on neutrophils.

## Discussion and conclusions

Our study indicated that the infiltration of neutrophils was markedly higher in peritumoural tissue compared with that in the actual tumour site in cases of HCC. Moreover, the number of neutrophils in the peritumoural tissue was negatively correlated with the number of T cells. In addition, a lower level of neutrophil infiltration and a decreased neutrophil-to-lymphocyte T cell ratio (pNLR) in peritumoural tissue were correlated with prolonged patient survival after surgical treatment. These findings suggest that patients with a high level of peritumoural neutrophil infiltration and pNLR require a much closer follow-up after surgery. We reported here that the co-inhibitory molecule PD-L1 was overexpressed on neutrophils from patients with HCC. The tumour microenvironment contributes to the up-regulation of PD-L1 expression on neutrophils both in vitro and in vivo. Although PD-L1 expression has been observed on neutrophils in patients with infectious diseases, such as patients who are infected with tuberculosis [28] and HIV [27], this is the first study that has suggested that this negative regulatory pathway is mediated by neutrophils in HCC. Further in vitro studies have confirmed that tumour-derived factors including TNF-α and GM-SCF contribute to the induction of PD-L1 expression on human neutrophils. PD-L1/PD-1 interaction plays critical role in tumour development. Taken together, our results suggest that peritumoural neutrophils may foster immune privilege and disease progression via the PD-L1/PD-1 pathway in hepatocellular carcinoma.

The prediction of survival is critical to the management of HCC. Our data suggest that the level of peritumoural neutrophil infiltration as well as the pNLR value could be used for the prediction of patient survival after surgery. Data from clinical studies have indicated the correlation between the presence of neutrophils and poor prognosis. An increased level of neutrophils in peritumoural tissue promotes angiogenesis at the edge of the tumour via the production of MMP-9 [7], which indicates the pro-tumour phenotype of neutrophils. Li et al. showed that the intratumoural neutrophil infiltration rather than the peritumoural neutrophil infiltration predicts patient prognosis [8]. Another study also suggested that an elevated NLR was significantly correlated with HCC recurrence after liver transplantation via an inflammatory tumour microenvironment generated by TAMs and IL-17-producing cells [13]. Alternatively, the phenotype and function of neutrophils are highly heterogeneous in the tumour milieu. A recent study showed that tumour- infiltrating neutrophils stimulate an anti-tumour response in T cells in early-stage lung cancer [30]. Our study indicated the prognostic value of peritumoural neutrophil infiltration. In addition, our results also demonstrated that the pNLR better predicts patient survival with a minimum *P* value than the number of infiltrating neutrophils, which indicates the regulatory function of neutrophils on adaptive immunity in the development of HCC. Despite the different conclusions of these studies, our study was in accordance with those from the two groups that found that neutrophils predominantly infiltrated the peritumoural tissues rather than the tumour site itself. This suggests that a high level of peritumoural-infiltrating neutrophils should not be ignored in the management of HCC.

A number of studies have indicated a prognostic value of a higher preoperative NLR in patients with HCC [12, 13, 31]. However, the mechanisms remain to be elucidated. A lower intratumoural CD66b^+^ neutrophil/CD8^+^ T cell ratio has been shown to be associated with prolonged RFS and OS in patients with HCC [8]. We also observed that the CD66b^+^ neutrophil/CD3^+^ T cell ratio in peritumoural tissue was significantly higher and a better predictor of patient survival than that in the tumour itself. The peritumoural site is a barrier to the migration and dissemination of tumour cells in the earlier stages of cancer development. On the contrary, the peritumoural site is often the favourable “special zone” for the dissemination of tumour cells due to angiogenesis and the immunosuppressive micro milieu; this milieu is characterised by the infiltration of multiple types of stromal cells including lymphocytes, TANs, TAMs, MDSCs, TAFs and vascular endothelial cells. The local tumour microenvironment contributes largely to the phenotypic and functional modification of neutrophils. A morphological analysis of the peritumoural marginal region has shown that this area is always rich in tumour-associated fibroblasts (data not shown) and immune cells, including neutrophils. Neutrophils accumulate in the tumour site due to the tumour microenvironment-derived cytokines and chemokines. For example, IL-17- producing T cells recruit neutrophils that then accumulate in the peritumoural region via the expression of chemokines by endothelial cells [7]. Chemokines like CXCL1 and CXCL5 plays a tumour-supportive role via the recruitment of neutrophils in HCC [10, 11]. Tumour stromal cells including fibroblasts, hepatic stellate cells and endothelial cells have been shown to produce inflammatory factors such as GM-CSF, TGF-β, VEGF, and CXC chemokines, among others, that are associated with the accumulation and polarization of neutrophils [32]. The present study showed that neutrophils predominantly infiltrated the peritumoural tissue, which does not exclude the role of tumour stromal fibroblasts in the peritumoural region.

Tumour-supportive neutrophils are rich in tumour-promoting products such as arginase, MMPs and VEGF. Cytokines and chemokines are an efficient impetus for the migration of neutrophils. For example, neutrophil infiltration is closely related to the presence of TGF-β in the tumour site [33] or to the presence of VEGF in the peritumoural tissue [7]. Inflammatory factors are not at equivalent levels in the intratumoural and peritumoural sites. Among the detected factors in this study, IL-1β, GM-CSF, G-CSF, TNF-α and IL-6 were increased significantly in tumour tissues compared with adjacent non-tumour tissues. In addition, the levels of GM-CSF, G-CSF, TNF-α and MCP-1 were significantly higher in peritumoural tissue than that in tumour tissue. MCP-1 also contributed to the migration and accumulation of myeloid cells. Our in vitro study showed that GM-CSF and TNF-α contributed largely to increased expression of PD-L1 on neutrophils. TNF-α produced by neutrophils contributes to the antitumour response in the early stages of tumour development. We hypothesised that TNF-α participates in the polarization of neutrophils in advance-staged tumours via the induction of PD-L1 expression, which is similar to the role of TGF-β. Our previous study indicated that TSN-treated stromal fibroblasts are the predominant originator of TGF-β, GM-CSF, TNF-α and IL-6 compared with TSN or naïve stromal cells (data not published). The interaction of PD-L1/PD-1 is part of a critical negative regulatory pathway of the immune response by T cells. Here, we showed PD-L1^+^ tumour-infiltrating neutrophils in HCC, especially along the edges of the tumours tissue. This confirmed the idea that stromal cells in peritumoural tissues contribute largely to the overexpression of PD-L1 on neutrophils.

Our study provides a possible explanation for the previous reports with regards to the prognostic implications of greater numbers of neutrophils and increased NLR. In addition, we demonstrated the critical role of the tumour milieu in the modulation of the phenotype and function of tumour-infiltrated neutrophils. Although neutrophils might be considered one of the targets of tumour therapy, future immune therapy strategies may harness the combinatorial targets.

## Additional files

Additional file 1:
**Table S1. **Patient characteristics. Additional file 2:
**Figure S1. **Morphological analysis of tumor-infiltrated neutrophils in peritumoral tissue. Additional file 3:
**Figure S2.** Inflammatory factors were detected in tumour culture supernatant and in control medium by a Luminex200 system.

## Abbreviations

TAMs: Tumour-associated macrophages
NLR: Neutrophil-to-lymphocyte cell ratio
DMEM: Dulbecco’s Modified Eagle’s Medium
FITC: Fluorescein isothiocyanate
HCC: Hepatocellular carcinoma
PerCP: Peridinin chlorophyll protein
PE: Phycoerythrin
PD-1: Programmed cell death 1
PD-Ls: Programmed cell death ligands
SDF-1: Stromal derived factor −1
MCP-1: Monocyte chemotaxis protein −1
TANs: Tumour-associated neutrophils
TAFs: Tumour-associated fibroblasts
TILs: Tumour-infiltrated lymphocytes
TLR: Toll-like receptors
TB: Tuberculosis
HIV: Human immunodeficiency virus
RFS: Recurrence-free survival
TSN: Tumor-supernatant
MMP: Matrix metalloprotease
ICC: Intrahepatic cholangiocarcinoma
