# Supplementary material for: Peritumoural neutrophils negatively regulate adaptive immunity via the PD-L1/PD-1 signalling pathway in hepatocellular carcinoma
Source: J Exp Clin Cancer Res. 2015 Nov 18;34:141. doi: 10.1186/s13046-015-0256-0 (PMC4652417; doi:10.1186/s13046-015-0256-0)
Supplement: Additional file 1: — Table S1. Patient characteristics. [file 13046_2015_256_MOESM1_ESM.docx]

**Peritumoral neutrophils negatively regulates the adaptive immunity via PD-L1/PD-1 signaling pathway in hepatocellular carcinoma**

**Author Names**

Gaixia He^1, 2^**^#^**, Henghui Zhang^1, 2, 4, 5^**^#^**, Jinxue Zhou^3^, Beibei Wang^4, 5^, Yanhui Chen^1, 2^, Yaxian Kong^4, 5^, Xingwang Xie^1, 2^, Xueyan Wang^1, 2^, Ran Fei^1, 2^, Lai Wei^1, 2^, Hongsong Chen^1, 2^*, Hui Zeng^4, 5^*

*1.Peking University People’s Hospital, Peking University Hepatology Institute, Beijing 100044, China*

*2.Beijing Key Laboratory of Hepatitis C and Immunotherapy for Liver Diseases, Beijing 100044, China*

*3.Department of Hepatobiliary and Pancreatic Surgery, Henan Tumour Hospital, Zhengzhou, Henan 450008, China.*

*4.Institute of Infectious Diseases, Beijing Ditan Hospital, Capital Medical University, Beijing 100015, China*

*5.Beijing Key Laboratory of Emerging Infectious Diseases，Beijing 100015, China*

**^#^**Gaixia He and Henghui Zhang contributed equally to this paper.

**Correspondence:**

Address reprint requests to Hongsong Chen and Hui Zeng

Hongsong Chen, MD, PhD,

Peking University People’s Hospital,

Peking University Hepatology Institute,

No.11 Xizhimen South Street, Beijing 100044, China.

Telephone: +86 10 88325724,

FAX: +86 10 68318386.

E-mail: chenhongsong@pkuph.edu.cn

Hui Zeng, MD, PhD,

Institute of Infectious Diseases,

Beijing Ditan Hospital, Capital Medical University

Beijing 100015, China.

Telephone: +86 10 84322621.

Fax: +86 10 84322606

Email: zenghui@ccmu.edu.cn

Supplementary Table 1. Patient characteristics

| Clinicopathological variables |  | Total |  | Peritumoral CD66b | | |
| --- | --- | --- | --- | --- | --- | --- |
|  |  | 149 |  | 120 (high) | 29 (low) |  |
|  |  | N (%) | *P* value | N (%) | N (%) | *P* value |
| Age (years) | ≤40 | 39(26.2) |  | 34(28.3) | 5(17.2) | 0.126 |
|  | 41-50 | 48(32.2) |  | 39(32.5) | 9(31.0) |  |
|  | 51-60 | 37(24.8) |  | 29(24.2) | 8(27.6) |  |
|  | >60 | 25(16.8) |  | 18(15.0) | 7(24.1) |  |
| Gender | Female | 17(11.4) |  | 13(10.8) | 4(13.8) | 0.745 |
|  | Male | 132(88.6) |  | 107(89.2) | 25(86.2) |  |
| HBsAg | Negative | 31(20.9) |  | 27(22.5) | 4(13.8) | 0.322 |
|  | Positive | 115(77.1) |  | 90(75.0) | 25(86.2) |  |
| HCV | Negative | - |  | - | - |  |
|  | Positive | 3(2.0) |  | 3(2.5) | - |  |
| Alcohol intake history | Never | 63(42.3) |  | 50(41.7) | 13(44.8) | 0.835 |
|  | Yes | 85(57.7) |  | 70(58.3) | 16(55.2) |  |
| Family history | No | 121(81.2) |  | 96(80.0) | 25(86.2) | 0.599 |
|  | Yes | 28(18.8) |  | 24(20.0) | 4(13.8) |  |
| AFP (nag/ml) | Mean | 1626.2 |  | 1276.3 | 3071.3 |  |
| Liver cirrhosis | No | 21(14.1) |  | 18(15.0) | 3(10.3) | O.767 |
|  | Yes | 128(85.9) |  | 102(85.0) | 26(89.7) |  |
| Tumour size (cm) | <2 | 5(3.4) |  | 4(3.3) | 1(3.4) | 0.842 |
|  | 2 to 5 | 35(23.5) |  | 27(22.5) | 8(27.6) |  |
|  | >5 | 109(73.1) |  | 89(74.2) | 20(69.0) |  |
| Tumour number | single | 92(61.7) |  | 75(62.5) | 17(58.6) | 0.832 |
|  | miltiple | 57(38.3) |  | 45(37.5) | 12(41.4) |  |
| portal vein tumour  thrombus | absent | 110(73.8) |  | 90(75.0) | 20(69.0) | 0.49 |
|  | present | 39(26.2) |  | 30(25.0) | 9(31.0) |  |
| Lympho metastasis | absent | 133(89.3) |  | 106(88.3) | 27(93.1) | 0.739 |
|  | present | 16(10.7) |  | 14(11.7) | 2(6.9) |  |
| Intrahepatic metastasis | absent | 131(87.9) |  | 108(90.0) | 23(79.3) | 0.121 |
|  | present | 18(12.1) |  | 12(12.0) | 6(20.7) |  |
| Distance metastasis | absent | 88(59.1) |  | 72(60.0) | 16(55.2) | 0.677 |
|  | present | 61(40.9) |  | 48(40.0) | 13(44.8) |  |
| TNM stage | I+II | 43(28.9) |  | 33(27.5) | 10(34.5) | 0.496 |
|  | III+IV | 106(71.1) |  | 87(72.5) | 19(65.5) |  |
| Overall survival  (median, months) | |  |  | 20.39 | 25.24 |  |
| Disease-free survival  (median, months) | |  |  | 3.31 | 6.5 |  |
