# Supplementary material for: Peritumoural neutrophils negatively regulate adaptive immunity via the PD-L1/PD-1 signalling pathway in hepatocellular carcinoma
Source: J Exp Clin Cancer Res. 2015 Nov 18;34:141. doi: 10.1186/s13046-015-0256-0 (PMC4652417; doi:10.1186/s13046-015-0256-0)
Supplement: Additional file 2: — Figure S1. Morphological analysis of tumor-infiltrated neutrophils in peritumoral tissue. [file 13046_2015_256_MOESM2_ESM.pdf]

Peritumoral neutrophils negatively regulates the adaptive immunity via PD-L1/PD-1 signaling pathway in hepatocellular carcinoma

Author Names

Gaixia He[1, 2#], Henghui Zhang[1, 2, 4, 5#], Jinxue Zhou[3], Beibei Wang[4, 5], Yanhui Chen[1, 2], Yaxian Kong[4, 5], Xingwang Xie[1, 2], Xueyan Wang[1, 2], Ran Fei[1, 2], Lai Wei[1, 2], Hongsong Chen[1, 2*], Hui Zeng[4, 5*]

1.Peking University People's Hospital, Peking University Hepatology Institute, Beijing 100044, China

2.Beijing Key Laboratory of Hepatitis C and Immunotherapy for Liver Diseases, Beijing 100044, China

3.Department of Hepatobiliary and Pancreatic Surgery, Henan Tumour Hospital, Zhengzhou, Henan 450008, China.

4.Institute of Infectious Diseases, Beijing Ditan Hospital, Capital Medical University, Beijing 100015, China

5.Beijing Key Laboratory of Emerging Infectious Diseases，Beijing 100015, China

[#]Gaixia He and Henghui Zhang contributed equally to this paper.

Correspondence:

Address reprint requests to Hongsong Chen and Hui Zeng

Hongsong Chen, MD, PhD,

Peking University People's Hospital,

Peking University Hepatology Institute,

No.11 Xizhimen South Street, Beijing 100044, China.

Telephone: +86 10 88325724,

FAX: +86 10 68318386.

E-mail: chenhongsong@pkuph.edu.cn

Hui Zeng, MD, PhD,

Institute of Infectious Diseases,

Beijing Ditan Hospital, Capital Medical University

Beijing 100015, China.

Telephone: +86 10 84322621.

Fax: +86 10 84322606

Email: zenghui@ccmu.edu.cn

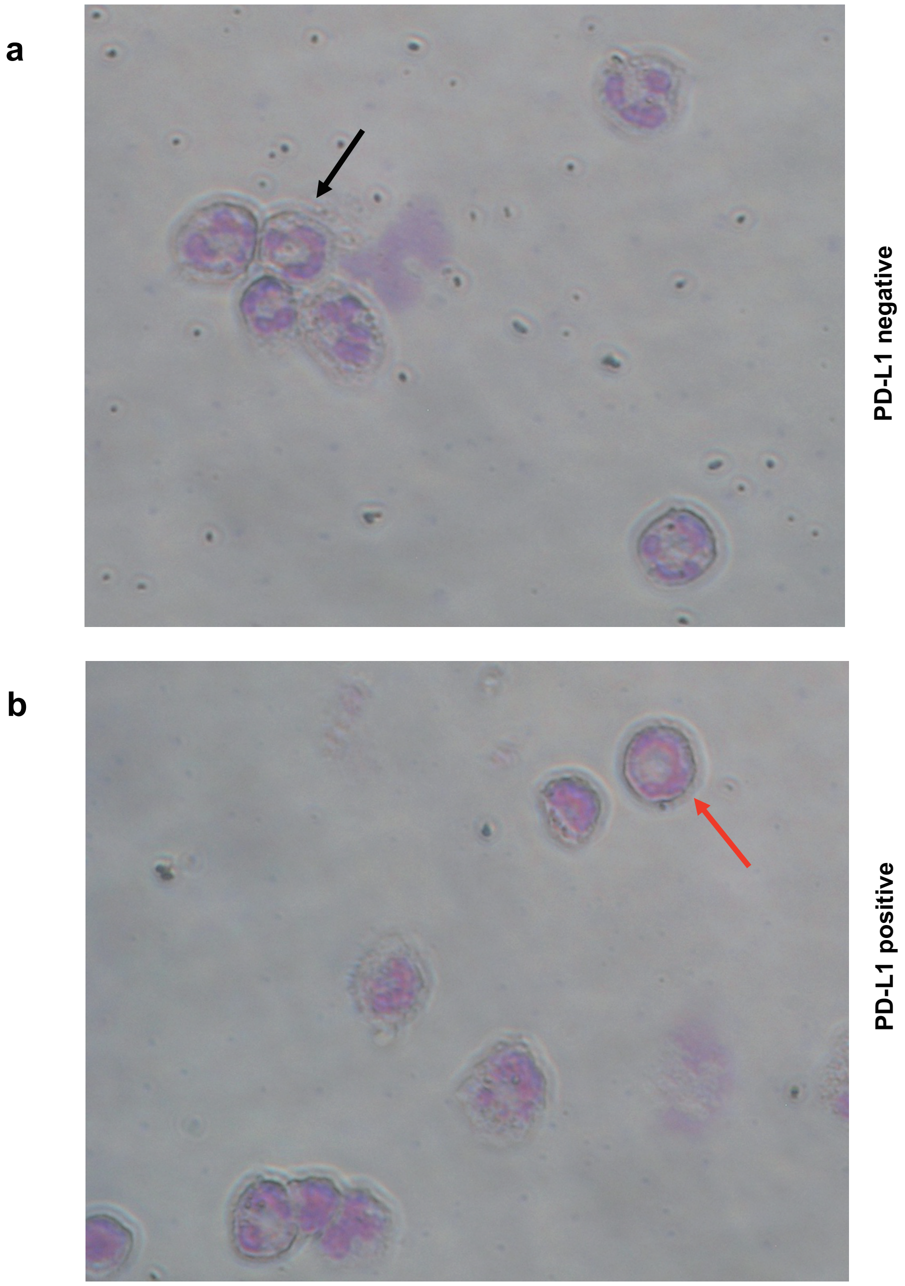

Fig. S1 Morphological analysis of tumor-infiltrated neutrophils in peritumoral tissue. (a) Cytospin images of the fraction of PD-L1^{+} neutrophils indicated segmented (black arrow) nuclei. (b) Cytospin images of the fraction of PD-L1^{-} neutrophils, including circle (red arrow) nuclei neutrophils.
